# The Role of Monoclonal Antibodies as Therapeutics in HPV-Related Head and Neck Cancers: An Updated Review

**DOI:** 10.3390/antib14020037

**Published:** 2025-04-24

**Authors:** Michael Zalin, Shaan Patel, Carter Coggins, Vikrant Rai

**Affiliations:** 1 College of Osteopathic Medicine of the Pacific, Western University of Health Sciences, Pomona, CA 91766, USA; michael.zalin@westernu.edu (M.Z.); shaan.patel@westernu.edu (S.P.); carter.coggins@westernu.edu (C.C.); 2 Department of Translational Research, Western University of Health Sciences, Pomona, CA 91766, USA

**Keywords:** human papillomavirus, head and neck cancer, immunotherapy, resistance, therapeutics

## Abstract

Background/Objectives: The increasing prevalence of human papillomavirus (HPV)-positive oropharyngeal squamous cell carcinoma (OPSCC) has necessitated a revaluation of therapeutic strategies. HPV-driven OPSCC differs from HPV-negative OPSCC due to its distinct molecular signatures, increased radiosensitivity, and better prognoses. However, despite these differences, treatment strategies have remained largely uniform, resulting in minimal reductions in morbidity and exposing HPV-positive patients to unnecessary toxicity. Monoclonal antibodies (mAbs) have become a promising therapeutic option due to their ability to target treatment with fewer systemic side effects. Immune checkpoint inhibitors (ICIs) such as pembrolizumab have shown efficacy in enhancing the immune response against tumors, while EGFR inhibitors like cetuximab offer an alternative modality. Current clinical trials aim to refine dosing regimens and identify combination strategies that may enhance therapeutic outcomes. Results: Despite promising evidence, several challenges hinder the widespread adoption of mAbs as a standard treatment for HPV-positive OPSCC in clinical practice. This review examines the current role of mAbs in HPV-positive OPSCC treatment, highlighting their limitations and future research directions. Conclusions: Further studies are needed to optimize patient selection, establish standardized treatment protocols, and investigate the long-term benefits of mAb-based therapies in this patient population.

## 1. Introduction

Head and neck cancer encompasses a broad range of malignancies, including those affecting the oral cavity, pharynx, and larynx. These cancers are responsible for nearly 900,000 cases and more than 400,000 deaths annually worldwide [1]. Alcohol and tobacco consumption are commonly identified as the primary risk factors for oropharyngeal squamous carcinoma (OPSCC); however, human papillomavirus (HPV) has emerged in recent decades as a distinct subset of these cancers. HPV-related head and neck cancers predominantly affect the oropharyngeal region, including the tonsils and base of the tongue, accounting for 31% of oropharyngeal cancers and 2% of the oral cavity and laryngeal cancers [2]. It has also been identified as the fastest-growing type of head and neck cancer in the United States [3]. The proportion of HPV-related OPSCCs has increased from 16% to more than 70% by the early 2000s [4] and surpassed cervical cancer to become the most common HPV-related cancer in the United States in 2012 [5,6]. Moreover, oral HPV DNA was found to be most prevalent in older males [7], likely due to the reactivation of latent infection and age-related immune compromise [8]. However, there is an increase in the cases of head and neck cancer in younger generations as HPV infections become more common [9], further demonstrating how HPV is becoming a dominant risk factor in OPSCCs.

The majority of HPV-positive OPSCC cases are attributed to HPV16 as it has high oncogenic potential by disrupting tumor suppressor pathways, notably the degradation of p53 [10]. Consequently, most research has focused on HPV16 and assumed that non-HPV16 genotypes behave similarly in OPSCC. Some studies have suggested that patients with non-HPV16 variants may have inferior survival outcomes [11], but the prognostic impact of individual HPV genotypes remains uncertain. However, HPV-positive OPSCCs demonstrate significantly improved survival outcomes as compared to patients with HPV-negative oropharyngeal cancers [12]. This better prognosis may be explained by fewer genetic alterations and increased radiosensitivity in HPV-positive tumors [13], which are associated with improved responses to therapy [14].

Although HPV-positive cases have improved disease-free and survival outcomes, treatment recommendations are similar to HPV-negative OPSCC [6]. Early stages are managed with radiation therapy (RT) or surgery, while advanced stages often use a multifaceted approach, which can include chemotherapy, radiation, and surgery [15]. Another method used to treat OPSCC is the use of monoclonal antibodies (mAbs). These mAbs are designed to specifically target a certain antigen, such as one found on cancer cells.

## 2. Materials and Methods

This manuscript is a narrative review. While we performed a literature search in PubMed and Google Scholar using keywords such as “HPV-positive OPSCC”, “epidermal growth factor receptor (EGFR) inhibitors”, and “monoclonal antibodies” to identify relevant studies, we did not conduct a full PRISMA-based systematic review nor undertake formal risk-of-bias assessments. Articles only in the English language were selected for this study. We aimed to review the literature for the past 10 years, but the articles from previous years have also been included for basic information.

## 3. Type of Monoclonal Antibodies

Depending on the source of mAbs, notably, there are four different ways mAbs can be made, and they are named based on their composition [16]. Murine mAbs are derived from mouse proteins by fusing B lymphocytes with immortal myeloma cells to produce immortal antibodies, but have a short half-life in humans [17]. Chimeric mAbs are made by combining the constant regions of human antibodies with the variable regions of mouse antibodies. The constant regions determine the antibody’s effector functions, and the variable regions responsible for antigen recognition regulate antigen specificity [18]. Humanized mAbs are mostly human proteins with only a small segment of mouse variable proteins attached to them, which reduces the antibody’s immunogenicity [19]. The development of these three classes of mAbs spans several decades, involving key breakthroughs in hybridoma technology and genetic engineering. The emergence of phage display technology allowed for the creation of the fourth mAb, the fully human mAbs, which have significantly improved safety, efficacy, and immunogenicity in humans because of their origin as entirely human [20]. Fully human mAbs are now widely used in the treatment of cancer, autoimmune, and infectious diseases.

Depending on the structure of antibodies, mAbs have been classified into naked mAbs, conjugated mAbs, and bispecific mAbs. The most common type of mAbs that are used to treat cancer is naked mAbs. They are characterized by working alone without any other radioactive substances or drugs attached. Naked mAbs are not exclusively derived from humans, as they can also include mouse antibodies. In most cases, naked mAbs attach to cancer cell antigens, but they can also attach to free-floating proteins or noncancerous cell antigens. Once bound to a cancer cell antigen, they can disrupt key pathways in cancer cell activity, such as those that cause uncontrollable growth [16,21]. Naked mAbs are also utilized in the treatment of HPV-positive OPSCC, notably ICIs and EGFR-targeting mAbs [22,23]. These mAbs represent a pivotal advancement in the treatment of HPV-positive OPSCC by utilizing their ability to precisely target cancer-related pathways and improve treatment efficacy while minimizing systemic toxicity as compared to traditional therapies. Conjugated mAbs are linked to chemotherapy drugs, radioactive particles, or other therapeutic agents to enhance their cytotoxic activity. These linked mAbs circulate throughout the body until they identify and bind to the target antigen while attempting to minimize damage to healthy tissues. Some limitations of these include potential off-target effects, risks of allergic reactions, and a high cost of production and administration [16,24,25]. Bispecific mAbs have two antigen-binding sites that allow them to target two different proteins or cells simultaneously. By targeting two different epitopes simultaneously, they can cause multiple independent or connected anti-tumor or physiological responses [26], which makes them ideal for cancer, autoimmune diseases, and inflammatory conditions. Bispecific mAbs are limited by potential off-target effects, manufacturing complexities, and instability, which impact their efficacy and safety [26].

## 4. Mechanism of Action of Monoclonal Antibodies

**Figure 1:**
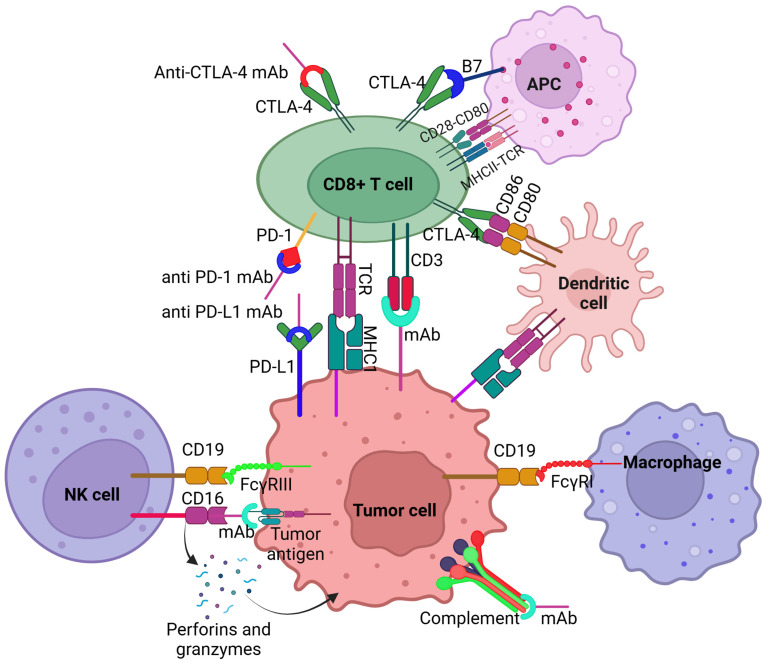
Mechanism of action of monoclonal antibodies (mAbs) and role of anti-PD-1/PD-L1 and anti-CTLA-4 antibodies in HPV-related head and neck cancer. FcγRI: Fc-gamma receptor I, NK cell: Natural killer cell, FcγRIII: Fc-gamma receptor III, PD-1: Programmed cell death protein-1, PD-L1: Programmed cell death ligand-1, MHCI: Major histocompatibility complex-1, CTLA-4: Cytotoxic T-lymphocyte associated protein 4, mAb: monoclonal antibody, CD: cluster of differentiation, APC: antigen-presenting cell.

The mechanism of action for mAbs includes activating the immune system, particularly T cells and natural killer (NK) cells. mAbs target and bind to antigens on cells, which leads to cell death by direct attack or membrane disruption, blocking of signaling pathways, or enhanced immune responses, all depending on the design and target of the antibody. NK cells recognize the antibody-coated tumor cells and release cytotoxic substances to kill the cells after the mAbs bind to the tumor cells. Activation of the complement system after binding of mAbs to tumor cells also contributes to the death of tumor cells. mAbs can also enhance T-cell activity and alter the immune environment to promote anti-tumor response. mAbs can also be engineered to deliver chemotherapy or radiotherapy directly to cancer cells specifically [27,28] (Figure 1). T cells can be activated with bispecific T-cell engagers and trispecific mAbs, where one arm of the mAb binds to CD3 on the T cell and the other arm binds to a tumor-specific antigen [29]. In HPV-positive OPSCC, this facilitates the interaction between T cells with the cancer cell through the overexpressed EGFR. T cells can also be activated by ICIs (Figure 1). Tumors often evade immune responses through the expression of programmed death ligand 1 (PD-L1) or inhibiting cytotoxic T lymphocyte-associated protein 4 (CTLA-4), which will inhibit T cell function [30]. This suggests that targeting PD-1/PD-L1 or CTLA-4 using mAbs will be effective in OPSCC tumors (Figure 1). Treatment with anti-PD-1 or anti-CTLA-4 mAbs in HPV-positive OPSCC can be effective as these tumors express PD-L1, and the mAbs will block these inhibitory signals and lead to enhanced T cell proliferation and cytokine release [31]. NK cells are an integral part of the innate immune system and can be activated by mAbs through antigen-dependent cellular cytotoxicity (ADCC). This occurs when the Fc region of the mAb binds the CD16 receptor on the NK cells, which stimulates them to release perforins and granzymes for the lysis of tumor cells [32]. NK cells are also activated by bispecific and trispecific NK cell engagers. One arm of the mAb binds to CD16 on the NK cell, and the other binds to the tumor antigen [33]. This increases NK cell-mediated cytotoxicity, specifically for tumor cells (Figure 1). Complement-dependent cytotoxicity is another key mechanism for mAbs to mediate the immune response to target cells. The Fc region of the mAb can recruit and activate the classic complement pathway through the binding of C1q [34]. This triggers the complement cascade and forms the membrane attack complex, which perforates the target cell membrane, leading to osmotic lysis and cell death [35] (Figure 1).

## 5. Molecular Mechanisms Underlying HPV-Positive and HPV-Negative Carcinomas

HPV+ and HPV− tumors arise from distinct carcinogenic pathways. HPV+ has virological origins for HPV+ cancers, and HPV− originates from exogenous chemical exposures, such as tobacco and alcohol, for HPV− cancers. For HPV− cancers, the potent combination of alcohol and products containing tobacco is a common driver of epigenetic changes that can directly result in the development of carcinoma. For example, increased production of nicotine-derived nitrosamine ketone, found as a direct consequence of tobacco use, can upregulate various oncogenic genes. This includes but is not limited to miRNA-21, miRNA-155, and miRNA-944, while also at the same time downregulating miRNA-422a, which acts as a tumor suppressor. A mutation of any of these miRNAs can cause a myriad of carcinogenic effects on a cell [36]. For example, mutation of miRNA-21 will upregulate uncontrolled cell growth by inhibiting phosphatase and tensin homolog (PTEN). Additionally, mutations to miRNA-155 cause the suppressor of Cytokine Signaling 1 (SOCS1) to be downregulated, which may ultimately lead to chronic inflammation, helping tumorigenesis. In particular, miRNA-944 will cause increases in proinflammatory cytokines and will upregulate signal transducer and activator of transcription 3 (STAT3), which has a direct contribution to tumor development by enhanced angiogenesis and metastasis. Additionally, there are tumor suppressor genes, such as TP53, that secondarily drive metastasis of HPV- tumors, which lead to greater genomic instability and therapeutic resistance. As a result, the highly mutagenic nature of HPV- tumors has a large contribution to the reduced efficacy of checkpoint inhibitors and further complicates treatment [37,38]. Genetic alterations lead to more hurdles in subsequent treatments, as well as reducing the efficacy of treatment options.

Alternatively, given the nature of HPV+ carcinomas relating to a virological origin and a deeper understanding of the molecular mechanisms at play, treatment is more predictable and usually offers better outcomes. Despite the many strains of HPV found, two strains in particular—16 and 18—are found in 99% of all HPV-related carcinomas [39]. HPV 16 will integrate into the genome through seven early genes (named E1–E7) and two late genes (L1–L2). The effect of E6 will cause degradation of p53 and E6-EAP-p53 complexes, while E7 will prevent the Retinoblastoma protein (Rb) from creating the Rb-E2F complex. Consequently, cell growth will continue beyond the G1-S checkpoint, into uncontrolled S-phase growth, ultimately inducing hyperplasia [40,41]. Some evidence suggests that E6 and E7 cause an upregulation of PD-L1, making it a potential predictive biomarker for treatment with mAb. This information can lead a physician toward the decision to use checkpoint inhibitor therapies, potentially improving treatment outcomes and creating a personalized therapeutic option in the HPSCC [42]. Further, silencing of E6/E7 oncogene in HPV+ tumors is associated with regulation of many microRNAs, including miR-17-5p, miR-186-5p, miR-378a-3p, miR-378f, miR-629-5p, and miR-7-5p (downregulation), and miR-143-3p, miR-23a-3p, miR-23b-3p, and miR-27b-3p (upregulation). These microRNAs regulate cell proliferation, cell senescence, and apoptosis, thus, tumor growth [43]. The change expressions of microRNA were found to be different in the cells and exosomes. Some microRNAs were found to be similar, while others had different expressions in intracellular vs. extracellular vesicles [44]. The expression profile of these microRNAs may help as a biomarker for disease progression and may also serve as a therapeutic target to reduce mortality as well as the development of novel therapeutics [45]. Further, an interaction between E6 and E7 oncoproteins with CAFs influences TME and regulates tumor progression, metastasis, and therapeutic resistance. Thus, the expression profile of CAFs may serve as a biomarker to guide treatment in HPV-related tumors [46,47,48].

In addition to checkpoint inhibition, there is an additional contributing factor: the suppression of endogenous immune reactions. HPV will downregulate major histocompatibility complex (MHC) class I receptors, which reduces the ability to be recognized by cytotoxic T-cells. This occurs through the E5 protein that binds to T-cell receptors, ultimately down-regulating MHC class I receptors and promoting tumor survival [49]. However, once these tumors are recognized by the host immune system through the infiltration of lymphocytes, there is strong immunogenicity. This creates an “immune-hot” tumor that is easier to target compared to HPV− tumors, which are “immune-cold”. This greater lymphocyte infiltration can be a future marker for mAb effectiveness, especially through the use of PD-1 inhibitors [50]. Dendritic cells also play a crucial role in developing adaptive immunity against HPV-related cancers by loading MHC class I and II receptors with HPV fragments to initiate an immune response. However, HPV evades immune detection by suppressing the secretion of key inflammatory cytokines, including interleukin (IL)-12, tumor necrosis factor (TNF)-α, and interferon (IFN)-γ, which are essential for recruiting dendritic cells. This suppression impairs T-cell activation, contributing to immune evasion and tumor persistence [51]. Given the current understanding of the checkpoints involved, this opens the door to potential therapeutic options, including the use of mAbs.

## 6. Monoclonal Antibodies in HPV-Related Head/Neck Tumors: Advantages

Treatment modalities for HPV+ OPSCC include primary chemoradiotherapy, transoral robotic surgery (TORS), and/or chemotherapy. In recent years, immunotherapy has become more common in use, however, the treatments often involve a combination of more than one modality [52]. TORS is a common first-line treatment for the benefit of head and neck cancers. A study evaluating the 2-year, actual, and disease-free survival rates reported a greater percentage of positive outcomes if patients had HPV+ tumors compared to HPV- tumors [53]. Despite the positive outcomes, there were cases of recurrence. Local regional recurrence has been found to range anywhere from 4 to 22%, and distal metastasis has been found in 2–22% of cases [54]. In some cases, the use of chemotherapy is a preferred option in favor of surgical intervention to preserve speech and swallowing functions that can be directly damaged by surgical interventions. In addition to TORS, radiotherapy is often used as a frontline treatment for HPV+ OPSCC. When using this arm of treatment in place of TORS, long-term survival outcomes showed a 5-year survival rate of 81%, which closely mirrors the effectiveness that is achieved through the use of TORS [55,56]. In some instances, a combination of chemoradiotherapy with surgery for OPSCC may be used, although, in a population-based study, concurrent chemotherapy did not appear to significantly affect the survival rate but resulted in worse long-term patient-reported outcomes. Thus, further research is warranted with a focus on the mechanism, prevention, and rehabilitation while treating patients with these long-term treatment toxicities [57].

However, in the case of recurrence after the use of frontline treatments, second-line treatments are immediately warranted. In such cases, the use of immunotherapy and mAbs becomes a viable treatment option. This type of treatment focuses on immune checkpoints. Certain types of tumors will downregulate the T-cell responses, allowing for uncontrolled growth. Hypopharyngeal squamous cell carcinoma (HPSCC) tumors utilize ICIs, which are mAbs that inhibit the inhibitor system [58]. In this way, scientists have been able to more effectively target tumors for degradation. One major checkpoint specifically targeted in these tumors is Programmed death receptor 1 (PD1). The PD1/PDL1 pathway is an important factor found in tumor immunosuppression, which causes inhibition of T lymphocytes, ultimately leading to tumor immune escape. It will create a complete absence of the immune response, including non-apoptosis from T cells, inhibition of CD4+ and CD8+ cells, as well as tumor-suppressing cytokines such as TNR, IFN-γ, and IL-2 [59]. Once the PD1’s pathway significance was identified, treatment options began to be developed. In 2019, the KEYNOTE-48 trial was completed, which specifically targeted the PD1/PDL1 pathway with promising results. The medication, pembrolizumab, was used in conjunction with chemotherapies and was found to have better results than other treatments that were approved for the treatment of HPSCC [60]. The data found in the KEYNOTE-48 trial demonstrated the value of mAbs as a viable first-line treatment. The overall survival of pembrolizumab alone vs. cetuximab with adjunct chemotherapy was 14.9 months vs. 10.7 months, and the use of pembrolizumab with chemotherapy vs. cetuximab with chemotherapy was 13.0 months vs. 10.7 months. There was no change in progression-free survival with the use of pembrolizumab alone or with conjunctive chemotherapy. No specific subgroup data for HPV-related carcinomas were reported at the end of KEYNOTE-48 [61] (Table 1). These are not the only mAbs that are currently used for the treatment of HPSCC. A detailed timeline has been discussed for anti-CTLA-4 and anti-PD-1 antibody targeting head and neck tumors [62]. Of note, beyond KEYNOTE-048, pembrolizumab and nivolumab have been approved for first-line treatment of HNSCC, including HPV+ OPSCC; however, mAbs like dostarlimab, atezolizumab, and durvalumab are being explored, and their first-line approval status in HPV+ OPSCC specifically is not yet as established [63,64,65]. Further, given the effectiveness of mAbs combined with favorable tolerance profiles, there are many ongoing clinical trials. Some of these mAbs include but are not limited to Durvalumab, a PD-1 inhibitor; Tremelimumab, a CTLA-4 inhibitor; and Peposertib, a DNA-PK inhibitor.

PD-1 inhibitors have emerged as highly effective agents in cancer therapy, making them a promising area for mAb research. Programmed Death 1 (PD-1) is an immune checkpoint found in T cells. Its normal function is to ensure that the normal immune response is not so strong as to destroy healthy cells in the body. However, they can be downregulated by the binding of their ligand, Programmed Death Ligand 1 (PD-L1), found on tumor cells. This allows the cancerous cell to proliferate unchecked, ultimately leading to a tumor. PD-1 inhibitors block this interaction, restoring T-cell function and enabling an anti-tumor immune response [66,67].

Dostarlimab is a PD-1 inhibiting mAb that is effective for the treatment of stage III or stage IV endometrial cancer. A Canadian study used dostarlimab in conjunction with carboplatin-paclitaxel in a head-to-head trial against a placebo and found that its use was able to provide statistically significant and clinically beneficial results in life expectancy as well as median progression-free survival [68]. Additionally, a 2022 phase 2 study involving 12 patients with locally advanced rectal cancer reported a 100% complete response rate following dostarlimab monotherapy, with no residual tumor detected via MRI. No patients had undergone conjunctive chemotherapy or radiotherapy [69]. While these results are preliminary, they do validate the potential therapeutic value of this treatment option. This trial was ongoing as of June 2024 and had expanded to include other types of cancer, including ovarian, melanomas, head and neck, and breast [70].

Nivolumab is another PD-1 inhibiting mAb used primarily in the treatment of malignant melanoma as well as other types of malignancies. It can be used on its own, however, it is commonly used in conjunction with another monoclonal antibody, ipilimumab, and was initially approved for therapeutic use in 2015 [71]. The effectiveness of treatment varies based on the tumor, but its use has been extended to other types of cancers, including hepatocellular carcinoma, classical Hodgkin lymphoma, small-cell lung cancer, and esophageal squamous cell carcinoma [71].

Pembrolizumab was the first PD-1 inhibitor approved for use in previously treated melanomas that were either metastatic or unable to be resected [72]. Like the other PD-1 inhibitors, its mechanism of action restores T-cell activity through the inhibition of PD-L1 and PD-L2 on tumor cells. While initially authorized for use in melanoma, it has demonstrated efficacy in the treatment of HNSCC. It can be used as a first-line therapeutic agent in certain circumstances as a single-agent therapeutic after the progression of the disease and failure of platinum-based therapies. It can also be used in conjunction with platinum-based therapies as a front-line treatment [73].

Cetuximab is a treatment option that is used in conjunction with radiotherapy when patients begin to develop a tolerance to radiotherapy when used by itself. Unlike the use of PD1 inhibitors found in pembrolizumab, cetuximab works by way of inhibiting the EGFR pathway [61]. Endothelial growth factor receptor (EGFR) works as a ligand-receptor system. Once it is initiated, it downregulates many miRNAs and upregulates new mRNAs, which control genes that determine the phenotype [74]. Cetuximab works by binding to the EGFR with greater affinity than its natural ligand, preventing the pathway from being initiated in the first place. In instances where tumor resection was necessary, patients were treated subsequently with cetuximab. It was found that the 24-month chance of tumor recurrence was 33.6% [75].

One of the main benefits of the use of mAbs in the use of HPSCC is the relatively few adverse effects that come with its use. Based on theory, the addition of antibodies is very favorable due to the relatively lower toxicity profile and pharmacokinetic interactions with the combined therapies. In real clinical use, this is confirmed to be the case. Previous studies have shown that mAbs in conjunction with chemotherapy did not worsen severe adverse reactions found with the use of other standalone modalities, indicating that the benefits of mAb addition are worthwhile. A recent clinical trial (NCT04533750) investigated the side effects and best dose of peposertib when given together with radiation therapy, and the results suggested that peposertib in combination with palliative RT is well-tolerated up to doses of 200 mg once daily [76].

The use of mAbs also has an additional use that has not yet been elucidated: the use of mAb to create a targeted response not by its own use but by delivering therapeutics directly. As of October 2024, there are a total of 15 ADCs approved for use as cancer treatments. While the concept behind it is good, there are some concerns with its use. There are several potential side effects and drug resistance noted with its use [77]. Despite this roadblock, there are many clinical trials ongoing to improve cancer outcomes with this methodology. At the time of this writing, clinicaltrials.gov revealed 200+ studies ongoing with the use of ADCs. This could be a potentially fruitful space for further treatment options. We will continue to monitor the progress made with interest. Further, many clinical trials, including early-phase studies and trials incorporating immunotherapy are exploring bispecific antibodies for HPV-positive OPSCC intending to improve treatment outcomes and minimize toxicity by targeting multiple cancer-related antigens and/or leveraging the immune system to fight cancer. Bispecific Antibody-Drug Conjugates (BsADCs) have been engineered to bind to two different targets simultaneously, often a tumor cell surface antigen and a component of the immune system, like T cells. A recent study by Dong et al. reported that BsADC targeting PD-L1 and B7-H3 enhances antitumor efficacy and promotes immune-mediated antitumor responses through immune checkpoint inhibition and promotion of immunogenic cell death [78]. Recruiting T cells near the tumor, followed by tumor cell death by activated T cells, may also be achieved by CD3 x EGFR bispecific antibodies and PD-L1-targeting ADCs, aiming for enhanced T cell responses. Bispecific antibodies, like Y111 (PD-L1 x CD3), work by bringing T cells into close contact with tumor cells and inducing their death, while ADCs target specific tumor cells with an antibody. Bispecific antibody Y111 targets CD3 on Vγ2Vδ2 T cells and PD-L1 on tumor cells to kill tumor cells [79]. Anti-CD3 x anti-EGFR bispecific antibody-armed activated T-cells (AATC) attach and selectively cross-link EGFR-expressing tumor cells and CD3-expressing T-cells, resulting in cytotoxic T-lymphocytes (CTLs) activation and selective cytotoxicity towards the EGFR-expressing tumor cells [80].

## 7. Resistance to Immunotherapy in HPV-Related Head and Neck Cancer

**Figure 2:**
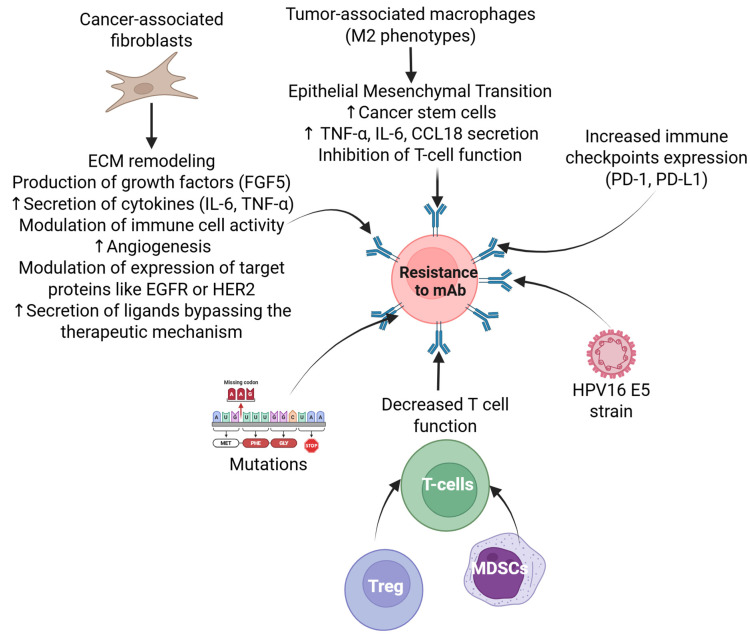
Mechanisms associated with immunotherapy resistance in HPV-associated head and neck cancer. ECM—extracellular matrix, IL6—interleukin 6, TNFα—tumor necrosis factor alpha, FGF5—fibroblast growth factor 5, EGFR—epidermal growth factor receptor, HER2—human epidermal growth factor receptor 2, CCL18—C-C motif chemokine ligand 18, PD-1—programmed cell death protein 1, PD-L1—programmed cell death ligand 1, HPV—human papillomavirus.

The presence of HPV16 E5 oncoprotein has also been associated with resistance to PD-L1 blockade, and tumors expressing HPV E5 can be targeted with rimantadine in HPV-related head and neck cancer [81]. Further, the presence of tumor microenvironment, which is less inflamed and more immunosuppressive due to the presence of myeloid-derived suppressor cells (MDSCs) and regulatory T cells (Tregs), suppresses T-cell function and promotes tumor growth in HPV-positive tumors compared to HPV-negative cancer, contributing to resistance to immunotherapy [82,83]. Resistance to immunotherapy may also be imparted by increased expression of immune checkpoints like PD-L1, which leads to T-cell exhaustion and reduced anti-tumor activity [84] (Figure 2).

Resistance may also be acquired after the initial response to treatment due to mutations in the tumor, changes in the tumor microenvironment, and the emergence of drug-resistant clones [81,84,85,86]. The presence of tumor-associated macrophages (TAMs), particularly the M2-like subtype, in HPV-related head and neck cancer is associated with resistance to immunotherapy and poor prognosis by promoting tumor progression and facilitating metastasis [46,87,88]. Further, the presence of cancer-associated fibroblasts (CAFs) and heterogeneity of CAFs may contribute to resistance to immunotherapy by interacting with tumor cells and influencing the tumor microenvironment. CAFs interact with tumor cells via the secretion of growth factors, cytokines, chemokines, and extracellular vesicles (EVs). CAFs may also contribute to increasing invasiveness and resistance to therapy by inducing epithelial-mesenchymal transition, suppressing immune response via the production of immunosuppressive molecules and recruiting immunosuppressive cells, and reprogramming metabolism in the tumor cells [89,90,91,92] (Figure 2).

Given these potential setbacks, treatment options must become available to bypass this resistance. There have been multiple trials and treatment options explored towards this end. One avenue has been ICI with the addition of varying nanoparticles. While the clinical efficacy of this has yet to be confirmed, there appears to be some potential. For example, gene silencing through RNA interference (RNAi) has been shown in HPV HNSCC cell lines to be able to significantly downregulate the E6 and E7 genes as well as upregulate anti-tumor p53 and Rb proteins in vivo and in vitro. At this point, however, the challenges presented with its use are the vehicle for delivery as well as finding ways to minimize its toxicity [93]. In addition to these challenges, another important one persists: this process acts as a knockdown for the genes. Constant activation is needed for full benefit, and while this may have its place in the future, it is not highly practical. Comparatively, CRISPR has shown some potentially profound benefits with its use. Using CRISPR-Cas 9, in keratinocytes that were transformed by HPV-16, there was a reduction of 5-fold in E7 gene levels [94]. This was conducted in cell lines, so the data is not conclusive, but it does show promise as a potential therapeutic in conjunction with mAb to circumvent resistance that develops.

Additional avenues have been explored, which include the combinatorial use of HPV therapeutic vaccines with the use of mAb, which have demonstrated some potential benefits and are currently being explored today. A study demonstrated that combinatorial treatments of an HPV E7 vaccine in conjunction with anti-CTLA-4 mAb were able to produce significant tumor regression in mouse models [95]. A recent Phase 1 study combined an HPV DNA vaccine with durvalumab in 12 patients. Minimal adverse effects were noted, and of the 11 study participants who were re-evaluated at the end of the trial, 9 of them had E6 or E7-related T-cell responses to the vaccine [96]. While it is far too early to declare these viable treatments, they do raise interest in the future directions of treatments.

Several pre-clinical combination therapies are being investigated to overcome resistance mechanisms in HPV+ HNSCC, including ICI + radiation, ICI + small molecules, and ICI + oncolytic viruses. These strategies aim to enhance the effects of ICI by addressing resistance mechanisms and inducing a stronger anti-tumor immune response. Jhawar et al., using a skin cancer mouse model, reported improved outcomes with a combination of oncolytic virus, radiotherapy, and ICI treatment via increased CD8+ T cell infiltration and IL-1α expression [97]. Dong et al. reported that combining ICI (pembrolizumab) with the oncolytic virus (Talimogene laherparepvec; T-VEC) may have significantly higher antitumor efficacy than either drug alone in head and neck cancer and pancreatic cancer [98]. Combining ICI with radiotherapy has shown promising results of enhanced immune response and tumor control by inducing immunogenic cell death and releasing tumor-associated antigens by radiotherapy further augmented by ICI [99]. Similarly, preclinical and clinical trials combining ICI with small molecules have shown promising results. For example, Sotorasib, a KRASG12C inhibitor, has shown promising results in combination with ICI to improve outcomes in KRAS-mutated tumors [62]. Further, combining ICI with radiotherapy has also shown promising results. Radiotherapy induces immunogenic cell death, releasing tumor-associated antigens that can be recognized by the immune system, and these responses are further enhanced by ICI by blocking immune checkpoints in head and neck cancer [99].

## 8. Challenges and Future Directions

At this point, numerous hurdles have yet to be overcome. Cost is a major barrier to the normalization of the use of mAbs [100]. For example, in the United States, pembrolizumab treatment may cost thousands of dollars [101]. While there are financial assistance programs available to help mitigate costs, these are not available to everyone and can lead to situations where access is untenable. This can also manifest in limited resources being available to lower-income areas, which further drives inequity in healthcare outcomes. Additionally, entire mAbs require a mammalian expression system to create a functioning antibody that can create a clinical effect. For this reason, companies are not able to use lower organisms to create these antibodies, which makes the synthesis of these antibodies economically infeasible in large quantities [102]. Despite the potential use as a frontline treatment and some promising results, mAbs are often used in conjunction with other therapeutic modalities as second-line treatments due to the current levels of effectiveness demonstrated. Despite the promise of mAB in OPSCC treatment, one major limitation is the lack of clear cell-surface targets. The effectiveness of mAbs relies on the high binding affinity to the antigen of choice, and given the lack of specificity available, this makes them not a viable first-line treatment. Additionally, in some circumstances, tumors develop resistance to the use of mAbs [103], which renders the use of the antibodies useless.

**Figure 3:**
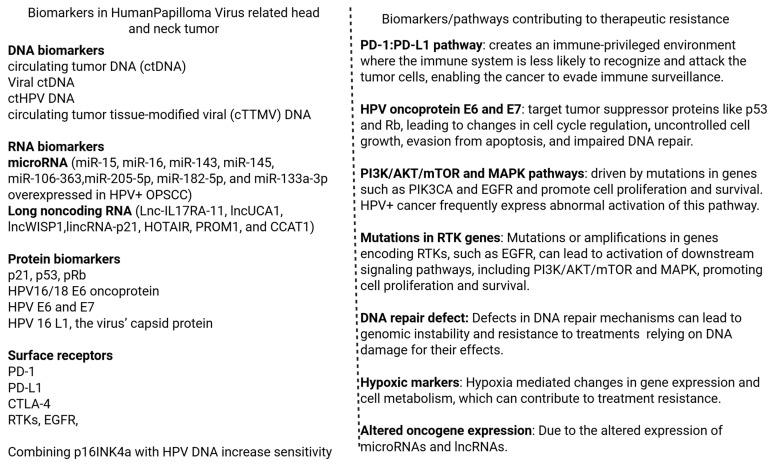
Biomarkers and their interaction for therapeutic resistance in HPV-positive head and neck cancer. Resistance to treatment can arise through various pathways, often involving the evasion of immune responses and alterations in cellular pathways [114,115,116,117,118,119,120].

Despite these challenges, mAbs represent a significant step toward personalized medicine. Using a patient’s genome, scientists would be able to individualize treatment options to more effectively target their exact disease [104]. Personalized medicine has long been a promising avenue in oncology, and the ability of mAbs to selectively bind tumor-specific markers could improve treatment efficacy [105]. While it is ideal to assume that everyone who has cancer would be able to receive the treatment that is needed, this is not always the case. A retrospective cohort study in Texas found that Medicaid and uninsured patients were significantly less likely to receive mAb therapy, despite its clinical relevance [106]. This was found to be the case among non-white patients, especially in regions with lower socioeconomic and educational levels [107]. Beyond accessibility, the effectiveness of mAb therapy depends on a patient’s immune profile. It was found that patients who expressed higher levels of CD8+ T cells demonstrated better responses to the use of mAbs. Conversely, patients who had high levels of immunosuppressive cells, such as regulatory T cells (Tregs) and Myeloid-derived suppressor cells (MDSCs), demonstrated poorer responses to the use of mAbs [108]. While expanding access to mAb therapy is crucial, its utility is ultimately dependent on whether a patient’s immune system can respond effectively. Addressing these barriers through improved biomarker identification (Figure 3), cost-reduction strategies, combination therapies, using improved and novel immunotherapies, targeted HPV-related mechanisms, and equitable healthcare access will be essential for optimizing the role of monoclonal antibodies in cancer treatment [31,84,85,109].

One potential solution to these disparities would be to improve access to biosimilars. Due to existing patents on certain mAbs, there are procedural and legal steps that must be taken to prevent any delays in the process of creation. As such, progress is slower than preferred, but it is ongoing [110]. One such study does exist, validating the effects of biosimilars. Galvao et al. conducted a review that demonstrated similar efficacy of biosimilars to their original formulas [111]. The financial costs were not detailed, but improved access to these medications is a step in the right direction. Ideally, the perfect solution would be to minimize and eliminate any illness from occurring in the first place. This could be helped by continued vaccination efforts with Gardasil 9. Evidence from clinical trials demonstrated up to 91–100% vaccine efficacy in women under 26 years of age and 83% in women over 26 years old [112]. According to the CDC, as of 2018, only 39% of adults aged 18–26 had received at least one dose of Gardasil 9 [113]. While this data may have changed at the time since its initial release, it does demonstrate the potential for further uptake. This would not only improve the health outcomes of those potentially infected, but it would also decrease the public health burden and costs associated. These strategies may promote and help in personalized treatment because biomarkers will help in early diagnosis, determining resistance to treatment, and deciding treatment strategies (Figure 3).

Finally, it is important to understand how the knowledge of biomarkers and available monoclonal antibodies should be used in clinical practice. A clinical algorithm integrating PD-L1 expression, tumor-infiltrating lymphocyte (TIL) density, and HPV subtype can guide monoclonal antibody (mAb) therapy by stratifying patients into groups with different expected responses to PD-1/PD-L1 blockade. PD-L1 expression is measured by immunohistochemistry, and it is a key predictor of response to anti-PD-1/PD-L1 mAbs. TILs, particularly CD8+ T cells, infiltrate TME, and their higher density suggests a more robust immune response, which can be further enhanced by PD-1/PD-L1 blockade. HPV-positive tumors, particularly in oropharyngeal squamous cell carcinoma (OPSCC), often exhibit higher PD-L1 expression and TIL density compared to HPV-negative tumors, and this difference is due to the immune system’s response to HPV infection and the tumor’s attempt to evade immune surveillance. By combining these factors, a clinical algorithm can be developed to guide mAb therapy. For example, patients with high PD-L1 expression, high TIL density, and HPV-positive tumors may be considered more likely to benefit from anti-PD-1/PD-L1 mAbs compared to those with low PD-L1 expression, low TIL density, and HPV-negative tumors [121,122,123,124,125]. It should also be noted that the CD8+/Treg ratio and IFN-γ expression can be more informative than PD-L1 alone for treatment stratification, in the context of ICI therapies. The CD8+/Treg ratio, reflecting the balance between cytotoxic and regulatory T cells, can be a more comprehensive indicator of immune response than PD-L1 expression alone, while IFN-γ expression can also provide valuable insights into treatment response [126,127].

## 9. Conclusions

Despite the promise of mAbs, most HPV+ OPSCC patients still receive chemoradiation. Monoclonal antibodies signify a powerful advancement in the treatment of HPV-related head and neck cancers, providing targeted and effective alternatives to conventional therapeutics. Immune checkpoint inhibitors and EGFR targeting agents, in particular, have shown great promise, despite challenges such as treatment resistance and accessibility remaining. However, the role of mAbs is currently limited to recurrent/metastatic settings, and future directions involving biomarker-based de-escalation strategies should be considered. Further research and clinical trials improving efficacy are warranted.

## Figures and Tables

**Table 1:** Outcomes of Pembrolizumab, Nivolumab, and Cetuximab in KEYNOTE-48.

mAb	Overall Response Rate	Progression-Free Survival	Overall Survival
Pembrolizumab	35.6% combination vs. 36.3% standard care	No difference	12.3 months vs. 10.3 months
Nivolumab	13.3% in the nivolumab group vs. 5.8% in the standard therapy group	19.7% vs. 9.9% at 6 months	7.5 months vs. 5.1 months
Cetuximab	22.9% in cetuximab + Irinotecan vs. 10.8% with monotherapy	66% vs. 58% at 6 months	8.6 months vs. 6.9 months

## Data Availability

Not applicable.

## Abbreviations

The following abbreviations are used in this manuscript:

CTLA-4	Cytotoxic T lymphocyte-associated protein 4
CAFs	Cancer-associated fibroblasts
EGFR	Epidermal growth factor receptor
HPV	Human papillomavirus
ICI	Immune checkpoint inhibitor
IL	Interleukin
IFN-γ	Interferon gamma
mAbs	Monoclonal antibodies
MHC	Major Histocompatibility Complex
MDSC	Myeloid-derived suppressor cells
NK cells	Natural killer cells
OPSCC	Oropharyngeal squamous cell carcinoma
PD-L1	Programmed death ligand 1
PTEN	Phosphatase and tensin homolog
SOCS1	Suppressor of Cytokine Signaling 1
STAT3	Signal transducer and activator of transcription 3
TNF-α	Tumor necrosis factor alpha
TAMs	Tumor-associated macrophages
